# Prenatal Exposure to Organophosphate Pesticides and Neurobehavioral Development of Neonates: A Birth Cohort Study in Shenyang, China

**DOI:** 10.1371/journal.pone.0088491

**Published:** 2014-02-13

**Authors:** Ying Zhang, Song Han, Duohong Liang, Xinzhu Shi, Fengzhi Wang, Wei Liu, Li Zhang, Lixin Chen, Yingzi Gu, Ying Tian

**Affiliations:** 1 Department of Epidemiology, Public Health School, Shenyang Medical College, Shenyang, China; 2 Department of Obstetrics, Central Hospital Affiliated to Shenyang Medical College, Shenyang, China; 3 School of Medicine, Shanghai JiaoTong University, Shanghai, China; Indian Institute of Toxicology Reserach, India

## Abstract

**Background:**

A large amount of organophosphate pesticides (OPs) are used in agriculture in China every year, contributing to exposure of OPs through dietary consumption among the general population. However, the level of exposure to OPs in China is still uncertain.

**Objective:**

To investigate the effect of the exposure to OPs on the neonatal neurodevelopment during pregnancy in Shenyang, China.

**Methods:**

249 pregnant women enrolled in the Central Hospital Affiliated to Shenyang Medical College from February 2011 to August 2012. A cohort of the mothers and their neonates participated in the study and information on each subject was obtained by questionnaire. Dialkyl phosphate (DAP) metabolites were detected in the urine of mothers during pregnancy to evaluate the exposure level to OPs. Neonate neurobehavioral developmental levels were assessed according to the standards of the Neonatal Behavioral Neurological Assessment (NBNA). Multiple linear regressions were utilized to analyze the association between pregnancy exposure to OPs and neonatal neurobehavioral development.

**Results:**

The geometric means (GM) of urinary metabolites for dimethyl phosphate (DMP), dimethyl thiophosphate (DMTP), diethyl phosphate (DEP), and diethyl thiophosphate (DETP) in pregnant women were 18.03, 8.53, 7.14, and 5.64 µg/L, respectively. Results from multiple linear regressions showed that prenatal OP exposure was one of the most important factors affecting NBNA scores. Prenatal total DAP concentrations were inversely associated with scores on the NBNA scales.?Additionally, a 10-fold increase in DAP concentrations was associated with a decrease of 1.78 regarding the Summary NBNA (95% CI, −2.12 to −1.45). And there was an estimated 2.11-point difference in summary NBNA scores between neonates in the highest quintile of prenatal OP exposure and the lowest quintile group.

**Conclusion:**

The high exposure of pregnant women to OPs in Shenyang, China was the predominant risk factor for neonatal neurobehavioral development.

## Introduction

As one of the largest developing agriculture countries, China must manage to maintain and increase crop yields from year to year. Because of this, pesticides are widely used in agriculture. The annual application of synthetic pesticides on food crops in China exceeds 300,000 tons and the average amount of the pesticides used in one field unit in China is more than 2.5-5 fold higher than the global average [1]. Even though the Chinese government emphasizes and promotes the reduction of pesticide use in agriculture, data from Chinese Agricultural Ministry for 2006–2010 showed that the annual pesticide consumption has not decreased. Along with the wide-spread use of highly toxic organophosphate pesticides (OPs) (with potent toxicity to insects, relatively low costs and the decreased likelihood for pest resistance [2] ), OPs account for more than 1/3 of all insecticide use in China.

As a result of the heavy use of OPs in agriculture, more than 10 percent of fruits, vegetables, and cereal grains grown in China contain pesticide residues exceeding the national safety standard [3], [4]. For several middle toxicity Ops, such as chlorpyrifos and malathion, their residues mostly are detectable in vegetables [5]–[7]. Moreover, some highly toxic OPs such as parathion and methamidophos, which have been banned in China since 2007, have still been detected in vegetable samples through routine monitoring by the Ministry of Agriculture [8]. Besides raw foods treated by OPs, contaminated drinking water, dust, and spray drift commonly contribute to OPs exposure among the general population [9]. However, to our knowledge, it is uncertain to know the exposure level to OPs and the effect on the health of the general population in China.

OPs are also likely to be neurotoxic to humans, possibly by utilizing similar mechanisms that target the nervous system of insect pests. This concern is of particular relevance to the developing human brain, which is inherently much more vulnerable to injury caused by toxic agents than adult brains [10]. Because OPs can potentially cross the placenta, fetuses face the risk of *in utero* exposure to OPs. Together with lower levels of detoxifying enzymes deactivating OPs in fetuses than those in adults, fetus development could be severely affected by OPs [11], [12].

In animals, prenatal exposure to OPs may lead to embryo toxicity and developmental toxicity, including neurotoxicity [13]–[15]. The neurotoxic effects of high level acute poisoning caused by the enzyme acetylcholinesterase (AChE), for example, are well established. Thus, AChE is prohibited for use because it can impair the peripheral, autonomic, and central nervous system (the cholinergic crisis) [16], [17] [18]. The effects of long-term exposure to OPs resulting in acute toxicity are currently controversial. Several epidemiologic studies amongst agricultural workers found adverse neurobehavioral and neuropsychological effects, as well as other chronic occupational hazards, associated with long-term exposure to OPs [19]–[22]. An inner-city multiethnic cohort study, the Mount Sinai Children’s Environmental Health Center (New York City) Survey, showed that prenatal exposure to OPs (through indoor pesticides use) was associated with anomalies in primary reflexes of neonates, and even had long-term adverse effects on neurodevelopment in young children [23], [24]. While the studies from the cohort of mothers and children in an agricultural area of California, the Center for the Health Assessment of Mothers and Children of Salinas (CHAMACOS) cohort, showed that the prenatal exposure to OPs was adversely associated with attention span, intelligence, cognitive and other neurodevelopment problems in young children, but no associations were discovered in neonates [25], [26]. We speculate that the association of exposure to OPs during neurodevelopment is dependent on the level of exposure to OPs in different regions. Moreover, the effects of OP exposure among the general population are currently unknown.

In this study, we enrolled a cohort of pregnant mothers and their neonates in Shenyang, where OPs are often detected in vegetables and fruits [8], and investigated the maternal exposure to OPs during pregnancy by measuring urinary concentrations of dialkyl phosphate (DAP), which are the metabolites of OPs. The association between the prenatal exposure to OPs and neonatal neurobehavioral development was analyzed. We provide evidence for the effects of OPs on the general population and also provide valuable information in planning policies in the management of pesticides use and public health, especially in pregnant women and children.

## Methods

### Participants

Healthy pregnant women were recruited to participate in this study from the obstetric wards of the Central Hospital Affiliated with Shenyang Medical College in Shenyang from February 2011 to August 2012. The pregnant women who met the inclusion criteria were enrolled in this study: living in the city for over three years; must not present with hypertension, diabetes, thyroid hypofunction, heart diseases, and other chronic diseases prior to pregnancy; participants must be without heavy complications during gestation such as diabetes, anemia, and hypertension; no family or medical history of mental retardation, phenylketonuria, and Pompe’s syndrome for pregnant women or their spouses. Women were screened for eligibility, and enrolled if they consented to participate in the study. Infants with disorders associated with adverse neurodevelopment such as traumatic brain injury, meningitis, and severe neonatal illnesses were excluded. Of 307 eligible women, 249 pregnant women and their neonates were finally enrolled as participants in the study (response rate: 81.1%).

The study was approved by the Medical Ethics Committee of Shenyang Medical College. All the participants signed written informed consents before the study.

### Questionnaire

Face-to-face interviews were conducted with the women after delivery by a specially trained nurse. Information about each subject was obtained by questionnaire, which included data on demographic information, home characteristics, residential history, reproductive history, active and passive smoking, dietary habits, and alcohol and drug use. The use of pesticides during pregnancy was also questioned, including whether or not pesticides were used by pregnant mother or her family member, the types of pesticides, and frequency of use.

### Urine OP Metabolites Measurements

Urine samples were collected from each mother for the analysis of dialkyl phosphate (DAP) and other pesticide-specific metabolites. Specimens were aliquoted into pre-cleaned glass containers with Teflon-lined caps, bar coded and stored at −70°C. Samples were then shipped on dry ice to Shanghai Center for Disease Control (CDC) for analysis. Gas chromatography with flame photometric detection (GC-FPD) was used to analyze the DAP metabolites of OPs following the method of Wu et al [37].

Briefly, one milliliter of urine was pipetted into a 10 mL screw-top glass test tube, and 250 µL of a Dibutyl phosphate (DBP, 97% purity, obtained from Fluka and used as an internal standard (IS)) solution (4.0 mg/L) were added. Subsequently, 4mL acetonitrile was added and the sample was mixed. After vigorous mechanical shaking for 5min, the test tube was centrifuged (1200×g, 5 min, 25°C). The supernatant fluid containing DAP and DBP was transferred into a clean screw-top glass test tube. Sample volume was then reduced to 70°C to a volume of 0.5 mL with a gentle nitrogen stream. Residues were re-extracted with 3mL of acetonitrile that contained 1 g of Na_2_SO_4_, were shaken for 10 min and then centrifuged. The resulting extract was repeatedly evaporated at 70°C to 0.1–0.2 mL under a gentle stream of nitrogen. To the final extracts, 20 mg of K_2_CO_3_, and 25 µL of pentafluorobenzyl bromide (PFBBr) were added and heated at 50°C for 16 h to covert the phosphate acids to their pentafluorobenzyl (PFB) esters. The PFB-DAP derivatives were dissolved in 100 µL of toluene for injection into the GC-FPD.

Capillary gas chromatography with P-specific flame photometric detection (GC-FPD) after derivatization with PFBBr was used to determinate the DAP metabolites of OPs in urine. PFB derivatives were identified by GC-MS using a GC-FPD (Shimadzu GC-14A) in the electron ionization (EI) mode. The GC operating conditions were as follows: GC column, BP-10, 25 m×0.33 mm i.d., 0.25 µm film thickness (SGE, Australia). Column temperatures, 110°C (1min) –8°C/min –210°C (1min) –20°C/min –280°C (10 min). Injection port temperature, 280°C; detector temperature, 300°C. Nitrogen gas (99.99% purity) was used as carrier gas at a head pressure of 150 kPa. The detector gases used were air at 60 kPa and hydrogen at 80 kPa. The injection volume was 1.0 µL in the splitless mode (splitless time, 1min). A GC-MS (HP5890–5973) was used for structural elucidation of PFB derivatives of DAP. Injector conditions and chromatographic conditions used were the same as for the GC-FPD. Operating conditions were as follows: carrier gas, helium gas (high purity grade) at a flow rate of 1.0 mL/min; ion-source temperature, 250°C; electron ionization, 70 eV; interface temperature, 280°C.

Five nonspecific OPs metabolites were measured in each sample, including two dimethyl (DM) phosphate metabolites (DMP,DMTP) and three diethyl (DE) phosphate metabolites (DEP, DETP and DEDTP). To provide an overall assessment of precision, accuracy and overall reliability of the method, quality control (QC) samples were used as blank samples and inserted blindly among the study samples. The limits of detection (LOD) for the five metabolites were 2.0 µg/L for DMP and 1.0 µg/L for DMTP, DEP, DETP, and DEDTP, respectively. Individual metabolite levels below the LOD was assigned a value equal to the LOD divided by the square root of two, and this value was included in each sum.

Summed concentrations of DM and DE (the two dimethyl metabolites; DMP, DMTP) and three diethyl metabolites (DEP, DETP, DEDTP) were calculated to provide summary measurements for exposure. To compare with other studies, we converted each metabolite from its untransformed concentration (µg/L) to the corresponding molar concentration (nmol/L) according to the forum described elsewhere [28].

Metabolite concentrations were adjusted using creatinine concentrations to correct for variable urine dilutions in the spot urine samples. Creatinine concentrations in urine were determined using a commercially available diagnostic enzyme method (Vitros CREA slides; Ortho Clinical Diagnostics, Raritan, NJ).

### Assessment of Growth and Neurodevelopment Status of Newborns

Neonatal Behavioral Neurological Assessment (NBNA) was used as the measurement of neurodevelopment and was administered when the infants were 3 days old. NBNA was formulated by Bao et al. [29], based on the method of Brazelton and Amiel-Tison for behavioral neurological measurement in newborns as well as the experience in Chinese newborns, tested with distinct stability and reliability by several large cohort in China, and was observed to not be influenced by the geographic location and suitable for large surveys [30]–[32]. The NBNA assessed functional abilities, most reflexes and responses, and stability of behavioral status during the examination. It involves five scales: Behavior (six items), Passive Tone (four items), Active Tone (four items), Primary Reflexes (three items), and General Assessment (three items). Each item has three dimensions of score (0, 1 and 2). Twenty items are summarized in the Summary Score with a maximum score of 40. Neonates with Summary Score more than 37 were considered to be well developed, lower than 34 abnormal, and between those scores was the acceptable range. NBNA were conducted by two examiners who were trained in the training center of Beijing Children’s Hospital affiliated with the Capital Institute of Pediatrics in Beijing, China. Examiners were blinded with regard to exposure status when NBNA were carried out.

### Lead Concentration in Umbilical Cord Blood

Considering the potential confounding effects of other suspected neurotoxins such as lead, we measured the lead concentration in umbilical cord blood, which were collected at delivery. Graphite furnace atomic absorption spectrophotometer (MG2) was used to determine the concentration of lead and the LOD was 0.1 µg/L.

### Statistical Analysis

Data were inputted with Epidata 3.02 and analyzed with SPSS17.0 for Windows (IBM, New York, USA). The levels of urinary OP metabolites (DAPs) were calculated with and without adjustment for creatinine. DAP levels were normalized by logarithmic transformation. All analyses were conducted on non-creatinine adjusted values and models were rerun with creatinine adjusted values (ng/g creatinine) in sensitivity analyses.

Nonparametric tests (Kruskal-Wallis tests) were used to compare the levels of urinary OPs metabolites among the pregnant women. Pearson Correlation analysis was used to determine the relationship between maternal urinary OP metabolites during pregnancy and neonatal neurobehavioral assessments scores (NBNA scores).

Multiple stepwise linear regressions were applied to examine the association of maternal OP exposure during pregnancy and the neurodevelopment of neonates. First, the scores of five Scales and the Summary of NBNA were used as dependent variables respectively, and the concentrations (logarithmic transformation) of OPs urinary metabolites, total DAPs (or DMs and DEs) and as well as the potential prenatal risk factors, including maternal age, education, gestational age, prenatal BMI, neonatal bodyweight and the lead concentration in umbilical cord blood were used as independent variables. The statistical significance level was set at 0.10 for inclusion and 0.20 for exclusion of the independent variables in the stepwise process. Secondly, the potential dose-response relationships between maternal OP exposure and neonatal neurodevelopment outcomes were assessed. Subjects were grouped into five equally sized exposure groups with quintiles of urinary DAP concentrations, from the lowest exposure group (as reference group) to the highest OP exposure. Mean NBNA scores among each exposure group (second to fifth categories) were calculated by the multiple regression formula and compared with those in the reference group.

### Ethical Statement

The study protocol was approved by the Medical Ethics Committee of Shenyang Medical College. Written informed consents were obtained from all the mothers for present study.

## Results

### General Characteristics of the Participants

The characteristics of the participants are listed in Table 1. The average age of the pregnant women and their average prenatal BMI was 28.9±3.2 years old and 22.5±4.5 kg/m^2^, respectively. About 51% of the subjects had university or higher education background and about 86% was primiparous. Over 68% of the pregnant women consumed vegetables or fruit every day. The average gestation of the women at delivery was 38.9±1.0 weeks. The average birth weight and height of the neonates were 3500.0±462.2 g and 50.9±1.9 cm, respectively. Mean Summary Score of NBNA of the newborns was 37.6±1.6 and the individuals over 37 scores occupy 96% of the whole new babies.

**Table 1:** Demographic and Exposure Characteristics for 249 pairs of the pregnant women and neonates in Shenyang, China.

		N	Percentage
Age(year)			
18–24		23	9.2
25–29		127	51.0
30–34		91	36.6
≥35		8	3.2
Parity			
0		207	83.1
>1		42	16.9
Education			
Junior and Secondary school		35	14.1
High school		64	25.7
College and postgraduates		150	60.2
Resident			
urban		217	87.1
rural		32	12.9
Occupational exposure to pesticides before pregnancy			
yes		2	0.8
no		247	99.2
Occupational exposure to pesticides during pregnancy			
yes		1	99.6
no		248	0.4
Using insecticides in household during pregnancy			
no		124	49.8
rarely		76	30.5
often		49	19.7
Passive smoking during pregnancy			
rarely		190	76.3
often		32	12.9
Always		17	6.8
Vegetables consumed weekly during pregnancy			
1–3times		23	9.2
4–6times		68	27.3
daily		158	63.5
Fruit consumed weekly during pregnancy			
1–3times		22	8.8
4–6times		70	28.1
daily		157	63.1
Monthly household income (RMB)			
<1000		76	30.5
1000∼3000		94	37.8
3001∼5000		61	24.5
>5000		18	7.2
Newborns sex			
Male		138	55.4
Female		111	44.6
Birth weight(g), mean(sd)		3500.44 (462.24)	
Body length(cm), mean(sd)		50.94 (1.92)	
Length of gestation(week), mean (sd)		38.7 (0.9)	
NBNA total score	<37	10	4.0
	≥37	239	96.0

### Organophosphate Pesticide Metabolites Level in Urine

OP urinary metabolite levels of the study sample both adjusted and not adjusted for creatinine are shown in Table 2. The OP metabolite concentrations higher than the LOD ranged from a low of 6.8% for DEDTP, to a high of 95.58% for DEP. The maximum value without creatinine adjustment was 334.02 µg/L for DMP and 167.06 µg/L for DEP, respectively. The maximum value with creatinine adjustment was 453.04 µg/g for DMP and 305.92 µg/g for DMTP, respectively. The geometric mean (GM) values without creatinine adjustment for DMP, DMTP, DEP and DETP levels were 18.03 µg/L, 8.53 µg/L, 7.14 µg/L and 5.64 µg/L, respectively. The GM for DEDTP levels was not calculated because of the low detection frequency. The creatinine-adjusted GMs for DMP, DMTP, DEP, DETP and DEDTP levels were 24.02 µg/g, 11.29 µg/g, 9.49 µg/g, 7.58 µg/g and 0.46 µg/g, respectively.

**Table 2:** Detection Frequency, Creatinine Unadjusted and Adjusted Geometric Mean, Range and Percentile of organophosphate pesticides urinary metabolites in 249 pregnant women in Shenyang, China.

	DM				DE						
	DMP	DMTP			DEP			DETP		DEDTP	
Detection rate (%)	94.78	83.94			95.58			88.76		6.8	
*Unadjusted*											
GM	18.03	8.53		7.14				5.64		-	
25th	7.83	3.4		3.54				2.34		LOD	
50th	24.04	11.84		5.42				7.04		LOD	
75th	39.43	15.67		17.17				13.55		LOD	
90th	94.18	47.48		42.32				29.15		LOD	
Range	<LOD∼334.02	<LOD∼137.95		<LOD∼167.06				<LOD∼133.00		<LOD∼6.61	
*Creatine-adjusted*											
GM	24.02	11.29		9.49				7.58		0.78	
25th	9.72	4.07		4.29				2.93		0.46	
50th	26.86	13.39		8.58				8.2		0.77	
75th	60.1	29.49		20.45				19.95		1.34	
90th	151.25	61.83		53.02				44.74		2.75	
Range	0.19∼453.04	0.10∼305.92		0.36∼125.09				0.32∼102.21		0.03∼15.41	

The median of urinary metabolites of OPs among different characteristic of the pregnant women were compared (Table 3). The median concentrations of DMs, DEs and DAPs of the pregnant women aged more than 30 years were significantly higher than those under 30 years (*P<0.05*); the median concentrations of DMs, DEs and DAPs of the pregnant women living in rural areas were slightly higher than those living in urban regions, but this did not reach statistical significance (*P>0.05)*. Pregnant women with a prenatal BMI higher than 28 had higher concentrations of DMs, DEs and DAPs than those with prenatal BMIs less than 28 (*P>0.05)*; the median of DMs, Des, and DAPs in the group of high level of passive smoking were higher than others, while there were no significant differences (*P>0.05).*

**Table 3:** Comparisons of urinary metabolites of OPs medians by population characteristic of pregnant women in Shenyang, China*.

characteristic		*n*	Metabolites		
			DMs	DEs	DAP
**Age**(year)	<30	150	29.61	214.34	45.80
	≥30	99	38.88	18.23	58.19
	***P*** value		0.004	0.04	0.013
**Education**	Junior and secondary school	35	31.31	14.85	46.58
	High School	64	34.97	18.73	60.56
	College and postgraduates	150	35.11	14.85	50.49
	***P*** value		0.503	0.564	0.823
**Residues**	Urban	217	32.75	14.85	47.19
	Rural	32	38.89	27.13	74.09
	***P*** value		0.199	0.038	0.055
**Prenatal BMI**	BMI<28	69	36.99	15.78	53.35
	BMI≥28	180	55.87	24.98	78.91
	***P*** value		0.19	0.66	0.46
**Passive smoking**	rarely	190	34.62	15.78	50.99
	often	32	31.21	13.07	46.91
	Always	17	67.71	21.95	108.50
	***P*** value		0.517	0.675	0.467
**Household income**(Yuan/Month)	≤3000	154	34.33	15.78	51.99
	>3000	95	34.85	14.85	48.85
	***P*** value		0.09	0.79	0.24
**Gestational Age**(week)	<37	8	25.76	14.85	40.61
	≥37	241	34.27	14.99	50.99
	***P*** value		0.97	0.82	0.95

*compared by Kruskal-Wallis test.

### Neurodevelopment of Newborns

In the study, 96% of neonates were considered well-developed from a neurodevelopmental level (NBNA Summary Scores≥37) and less than 1% abnormal (NBNA Summary Scores<34). The median NBNA Summary scores for male and female neonates did not differ and equaled 38 (interquartile range: 37–39). NBNA total scores, behavior scores, passive tone scores, and primary reflexes scores between male and female newborns were not significantly different (Z = −0.377, Z = −0.406, Z = −0.705, Z = −0.543, Z = −0.524, respectively; P>0.05). Almost all neonates (>99%) scored full marks in the scale of General Assessment, therefore this scale was not further analyzed in the study.

### Relationship between Prenatal OP Exposure and Neurodevelopment of Newborns

First, Pearson correlation analyses were used to explore the relationship between maternal urinary OP metabolites (logarithmic transformation) during pregnancy and neonatal neurobehavioral assessments scores (NBNA scores). There were significantly negative correlations between summary NBNA scores and the concentration of maternal DMs, DEs, DAPs during pregnancy (r = −0.510, r = −0.494, r = −0.561, *P*<*0.01*).

Multiple stepwise linear regression analyses were then used to examine the association between neonatal neurobehavioral development and maternal OP exposure (total DAP as the index) during pregnancy. Maternal urinary DAP concentrations measured during pregnancy revealed significant associations with poorer NBNA scores (without other prenatal confounding influences). The adjusted coefficients (β) (95% CIs) for NBNA scores compared to OP metabolite levels (DAP, DM, DE) are represented in Table 4. From the results, higher prenatal DAP concentrations were associated with lower scores in all NBNA scales, especially the Behavior Scale (β for a 10-fold increase in concentration = –0.65, 95% CI, −0.85 to −0.45, *P<0.01*). Moreover, a 10-fold increase in total DAP concentration was associated with a decrease of 1.78 in NBNA Summary scores (95% CI, −2.12 to −1.45). Urinary DM concentrations during pregnancy was also associated with poorer NBNA scores in Passive Tone, Active Tone, Primary Reflex scales and the Summary (*P<0.05*), although point estimates were slightly lower than for total DAP concentrations. While urinary DE concentrations were associated with poorer NBNA scores in Behavior scale and the Summary (*P<0.05*), they were not as highly correlated compared to total DAP and DM concentrations.

**Table 4:** Adjusted Coefficients (β) (95% CIs) on the Behavior, Passive tone, Active tone, Primary reflexes And Summary scores of NBNA for a Log10 Unit Increase in OPs Urinary Metabolites among the neonates in Shenyang, China.

	Summary	Behavior	Passive Tone	Active Tone	Primary Reflexes
***Total neonates***					
DAPs	−1.78 (−2.12,−1.45)	−0.65 (−0.85,−0.45)	−0.22 (−0.34,−0.10)	−0.48 (−0.66,−0.30)	−0.36 (−0.51,−0.21)
DM	−0.96 (−1.35,−0.57)		−0.22 (−0.33,−0.11)	−0.41 (−0.57,−0.29)	−0.30 (−0.44.−0.17)
DE	−0.88 (−1.30,−0.47)	−0.59 (−0.79,−0.40)			
***Boy neonates***					
DAP	−1.47 (−1.93,−1.01)	−0.50 (−0.76,−0.23)	−0.21 (−0.36,−0.02)	−0.46 (−0.72,−0.21)	−0.34 (−0.55,−0.13)
DM	−0.93 (−1.45,−0.40)		−0.19 (−0.35,−0.07)	−0.34 (−0.58,−0.11)	
DE	−0.61 (−1.15,−0.07)	−0.42 (−0.67,−0.17)			−0.28 (−0.48,−0.09)
***Girl neonates***					
DAP	−2.03 (−2.55,−1.52)	−0.84 (−1.15.−0.52)	−0.21 (−0.40,−0.02)	−0.51 (−0.76,−0.25)	−0.39 (−0.61,−0.17)
DM	−1.22 (−1.89,−0.55)		−0.18 (−0.35,−0.01)	−0.41 (−0.65,−0.18)	−0.34 (−0.54,−0.14)
DE	−0.98 (−1.58,−0.39)	−0.83 (−1.15,−0.53)			

Estimates were adjusted for maternal age, education, gestational age, prenatal BMI and the lead concentration in umbilical cord blood.

Variable coding: Gestational age (<37week = 1, ≥37week = 2); Maternal Age (<30 = 1, ≥30 = 2); Prenatal BMI (BMI<28 = 1, BMI≥28 = 2); Passive smoking (rarely = 1, often = 2, always = 3); Neonatal bodyweight (<3000 = 1,3000−3500 = 2; >3500 = 3).

**Figure 1:**
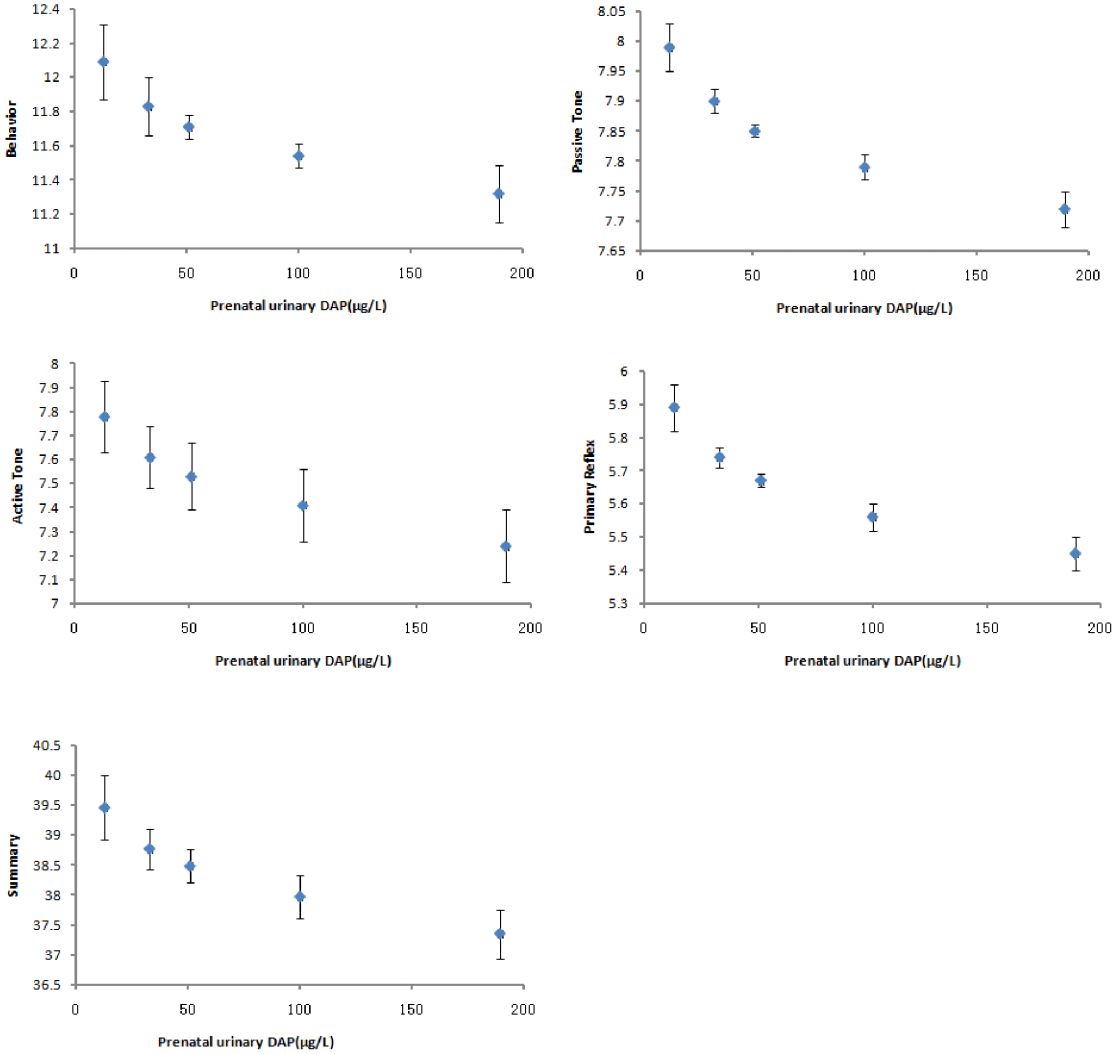
NBNA scores per quintile of prenatal urinary DAP concentration (Mean ± SD): Behavior, Passive Tone, Active Tone, Primary Reflex, and Summary. The medians (ranges) for DAP quintiles (µg/L) are as follows: first quintile, 13 (3–21); second, 33 (>21–42); third, 51 (>42–65); fourth, 100(>65–140); fifth, 189 (>140).

The NBNA scores in different maternal DAP concentration groups are shown in Figure 1. No evidence of departure from linearity in the relation between maternal DAP concentrations and NBNA scores was observed. There was a 2.11-point (in Summary) to 0.27-point (in Passive tone Scale) difference of estimate NBNA scores between neonates in the highest quintile of maternal prenatal DAP levels and those in the lowest quintile. Moreover, there is no difference between male and female neonates in that NBNA scores were negatively associated with the concentrations of maternal urinary OP metabolites (total DAPs) during pregnancy. For male neonates, a 10-fold increase in maternal pregnant urinary DAP concentrations was associated with a decrease of 1.47 points of summary NBNA scores. For female neonates, maternal pregnant urinary DAP concentrations were associated with poorer summary NBNA scores (β =  –2.03, *P* = 0.01). There were similar associations between urinary DM, DE concentrations and poor NBNA scores among male and female neonates, with slightly stronger estimates in girl neonates (Table 4).

The standard coefficients of the multiple stepwise linear analyses for NBNA scores and prenatal OPs metabolites (DAP) and other potential prenatal risk factors among the neonates are shown in Table 5. Prenatal OP exposure was evidently the strongest risk factor for lower NBNA scores, with the largest standardized regression coefficients (in absolute value) in all of the NBNA scales both in boys and girls (*P<0.05*). High levels of lead concentration in the umbilical cord blood and prenatal BMI were associated with poor NBNA scores (*P<0.05*). Maternal age, education, and neonatal bodyweight had no associations with NBNA scores in the present study as determined by multiple linear regression analysis *(P>0.05)*.

**Table 5:** Standardized regression coefficients from the Stepwise Linear Regressions for the NBNA scores and prenatal risk factors among the neonates in Shenyang, China (n = 249).

	Summary	Behavior	Passive Tone	Active Tone	Primary Reflexes
***Total neonates***					
DAP a #	−0.615^**^	−0.403^**^	−0.253^**^	−0.364^**^	−0.329^**^
Gestational Age	0.127^*^	0.170^*^			
Maternal Age					
Prenatal BMI		−0.122			
Passive smoking					
Blood Lead	−0.192^**^			−0.217^**^	
Neonatal bodyweight					
***Boy neonates***					
DAPs a #	−0.555^**^	−0.342^**^	−0.250^*^	−0.338^**^	−0.303^**^
Gestational Age	0.165	0.266^**^			
Maternal Age					
Prenatal BMI					
Passive smoking					
Blood Lead	−0.178^*^		−0.177	−0.198^*^	
Neonatal bodyweight					
***Girl `***					
DAPs a #	−0.672^**^	−0.503^**^	−0.236^*^	−0.394^**^	−0.361^**^
Gestational Age					
Maternal Age					
Prenatal BMI			−0.231^*^		
Passive smoking					
Blood Lead	−0.158			−0.240^*^	
Neonatal bodyweight					

Note: ^**^ P<0.01. ^*^ P<0.05.

a the concentration of maternal OPs urinary metabolites during pregnancy.

b the concentration of lead in umbilical cord blood of neonates.

# the value in regression was changed by the log10 transition.

Variable coding: Gestational age (<37week = 1, ≥37week = 2); Maternal Age (<30 = 1, ≥30 = 2); Prenatal BMI (BMI<28 = 1, BMI≥28 = 2); Passive smoking (rarely = 1, often = 2, always = 3); Neonatal bodyweight (<3000 = 1,3000–3500 = 2; >3500 = 3).

Point estimates for creatinine-adjusted DAP concentrations were similar to those for non-creatinine adjusted concentrations (data not shown).

## Discussion

Fetal exposure to OPs occurs because OPs can cross the placenta [33], [34]. Thus, fetuses are more vulnerable to OPs [35]. Exposure to low level of OPs can influence emotional behaviors [36] and neuronal cell development [37] through a variety of noncholinergic mechanisms such as disruption of various cellular processes [38], up regulation of serotonin neurotransmitters [39] and oxidative stress [40]. Since many OPs are lipophilic and rapidly metabolized in the human body by hydrolysis or oxidative desulfurization, dialkyl phosphate (DAP) metabolites in urine are often used as biomarkers to reflect the cumulative exposure to OPs in humans [41], [42]. In this study, we measured five non-specific metabolites of OPs in the urine, DMP, DEP, DMTP, DETP and DEDTP (referred to as DAPs) to assess the exposure to OPs.

In our study, the GM levels (nmol/L) of DAPs in pregnant women were 167.14 for DMP, 56.62 for DMTP, 44.68 for DEP, and 40.76 for DETP. The concentrations (GM) of DM and DE were 283.66 and 107.39 nmol/L, respectively. In a previous study from the Netherlands, the DAPs concentrations from 100 pregnant women were 79.9 for DMP, 60.9 for DMTP, 13.0 for DEP and 4.7 for DETP [43]. Data from the National Health and Nutrition Examination Survey (NHANES, 1999–2000) in the U.S showed that the median level of urinary DAP metabolites in pregnant women [44] was 72 nmol/L. The results from the Center for Health Assessment of Mothers and Children of Salinas (CHAMACOS) cohort study indicated that the average GM for prenatal maternal urinary DAPs, DM and DE are 109.0, 76.8 and 17.7 nmol/L [25]. One investigation in Shanghai also revealed high levels of urinary DAPs in pregnant women, similar to those found in our study [45]. The results above indicate that GM levels (nmol/L) of DAPs in China are significantly higher than those in the Western Counties, suggesting that people are exposed to higher levels of OPs in developing counties like China compared to those in developed countries. The reason for the high level of OP exposure in China is mainly due to the heavy use of OPs in agriculture, leading to high residues in food, especially in vegetables and fruits [27]. The pesticide (most of them are OPs) residues of vegetables and fruits in Chinese markets are easily detectable. Some of these fruits and vegetables show high levels of residues exceeding the national safe standard [6], even though the Chinese government announced in 2005 that the high toxic OPs such as dichlorvos, dimethoate, Parathion, methamidophos, monocrotophos and phosphamidon, are forbidden in use in agriculture. The mid-toxic pesticides such as chlorpyrifos and diazinon are always detected because they are commonly used for the control of pests in vegetables and fruits.

More than 68% of the pregnant subjects in this study consumed fresh vegetables and fruits every day during the pregnancy, suggesting that diet might be the primary source of OP exposure. One exception is that of one woman who was occupationally exposed to OPs (she is a pesticide production worker) before pregnancy. Investigations in the general American population [9] and in children [46] also concluded that diet exposure provided a vehicle for OPs to affect children as well as the general population. Compared to average individuals, pregnant women tend eat more food (especially more fresh vegetables and fruits) and drink more water than usual to obtain much more nutrition [47], therefore they face a higher risk of OP exposure.

Interestingly, we found a significant inverse association between maternal urinary metabolites of OPs (DM, DE and DAPs) during pregnancy and the NBNA scores of neonates. Among several potential prenatal risk factors for neonatal neurobehavioral development, including OPs exposure, maternal age, education, passive smoking, gestational age, prenatal BMI, blood lead concentration, maternal OP exposure (DAP concentrations) during pregnancy was the predominant factors to these scores. The estimate NBNA scores of neonates in the high-exposure group for the four scales and the sum of the scores were 0.27–0.77 points and 2.11 points higher than those in the low-exposure group, respectively. These results suggest that maternal exposure to OPs during pregnancy could influence neonatal neurobehavioral development. Our results are consistent with those from a pregnancy cohort study in New York City (the Mount Sinai Children’s Environmental Health Center), which concluded that prenatal OP metabolites in urine (primary DEs) are associated with an increasing number of abnormal primitive reflexes in neonates as evaluated by the Brazelton Neonatal Behavioral Assessment Scale [23]. Another longitudinal cohort in California, USA, from a cohort of the Center for the Health Assessment of Mothers and Children of Salinas (CHAMACOS) suggested that maternal pesticide exposure during pregnancy and not postnatal exposure is associated with poorer neonatal reflexes and long-term effects on children’s mental development at 2 years at age, poor attention skills at 5-years-old, as well as poor intellectual development in 7-year-olds are present [25], [26], [48], [49]. Although there are some differences between our study and the studies mentioned above, such as different measurements of neonatal neurodevelopment, similar conclusions were found: that the concentrations of maternal OP metabolites during pregnancy are inversely associated with the neonatal neurodevelopment.

The primary target of organophosphate insecticides is the enzyme acetylcholinesterase (AChE), which hydrolyses the neurotransmitter acetylcholine in both the peripheral and the central nervous system. In this study, we applied NBNA as the measurement of neonatal neurodevelopment. Based on the method of Brazelton and Amiel-Tison for behavioral neurological measurement in newborns, NBNA was utilized according to the condition of Chinese newborns [29]. NBNA was tested with distinct stability and reliability by several large cohorts in China, and was observed not to be influenced by the geographic location and suitable for large surveys [30]–[32]. In our study, NBNA was administered to the neonates when they were 3 days old to ensure the uniformity of the tests.

This study was not without limitations. For example, we measured the OP urinary metabolites at a single time point only prior to delivery. Urinary DAPs have a half-life of <24 h in humans, so the urinary concentrations reflect only recent exposure [50], [51]. The combination of the short elimination half-life and the episodic nature of exposures to OP pesticides results in a high degree of variability in urinary DAP concentrations. Secondly, urinary DAP concentrations cannot quantify exposure to a particular pesticide. Even though the serum OPs can evaluate the actual exposure to a particular pesticide, they are often undetectable. At present, urinary organophosphate metabolites and DAPs are still used as biomarkers of organophosphate pesticide exposure in many studies [25], [26], [43], [52], [53]. Nevertheless, our study provides important information on the adverse health effects of OPs on pregnant women and neonates. Further longitudinal studies with larger sample size and more representative samples will confirm the association of prenatal OPs and the neurodevelopment of neonates and young children.

## Conclusion

In summary, our study reveals that there exist high levels of exposure to OPs among pregnant women in Shenyang, China. This maternal exposure to OPs during pregnancy strongly associated with adverse neonatal neurobehavioral development. This study helps the local government to legislate against the abuse of OPs to improve the human health, especially the health of pregnant women and children.
